# Epidemiology and risk factors related to severity of clinical manifestations of COVID-19 in outpatients: A retrospective study in Haiti

**DOI:** 10.1371/journal.pone.0274760

**Published:** 2022-09-21

**Authors:** Mentor Ali Ber Lucien, Katilla Pierre, Gladzdin Jean-Denis, Jonas Rigodon, Caitlin M. Worrell, Alexia Couture, Aspen Flynn, Mauricio Cerpa Calderon, Luis Felipe Codina, Andrea S. Vicari, Samson Marseille, Koama T. Jean Baptiste, Bernadette Fouche, Gerard Joseph, Ito Journel, Kenold Rendel, Yoran Grant-Greene, Nadia P. Jean-Charles, Donald Lafontant, Senou Amouzou, Wilnique Pierre, Marie Greta Roy Clement, Stanley Juin, Jacques Boncy, Patrick Dely

**Affiliations:** 1 National Public Health Laboratory (LNSP)/ Ministry of Public Health and Population (MSPP), Port au Prince, Haiti; 2 Directorate of Epidemiology, Laboratories and Research (DELR)/MSPP, Port au Prince, Haiti; 3 Pan American Health Organization, World Health Organization (PAHO/WHO), Port-au-Prince, Haiti; 4 U.S. Centers for Disease Control and Prevention, Port-au-Prince, Haiti; 5 Division of Parasitic Diseases and Malaria, U.S. Centers for Disease Control and Prevention, Atlanta, Georgia, United States of America; 6 COVID-19 International Task Force Emergency Response Capacity Team, U.S. Centers for Disease Control and Prevention, Atlanta, Georgia, United States of America; 7 Pan American Health Organization, World Health Organization (PAHO/WHO), Washington, DC, United States of America; 8 Quisqueya University, Port-au-Prince, Haiti; 9 Ministry of Public Health and Population (MSPP), Port au Prince, Haiti; University of Zambia, ZAMBIA

## Abstract

**Background:**

Haiti’s first COVID-19 cases were confirmed on March 18, 2020, and subsequently spread throughout the country. The objective of this study was to describe clinical manifestations of COVID-19 in Haitian outpatients and to identify risk factors for severity of clinical manifestations.

**Methods:**

We conducted a retrospective study of COVID-19 outpatients diagnosed from March 18-August 4, 2020, using demographic, epidemiological, and clinical data reported to the Ministry of Health (MoH). We used univariate and multivariate analysis, including multivariable logistic regression, to explore the risk factors and specific symptoms related to persons with symptomatic COVID-19 and the severity of symptomatic COVID-19 disease.

**Results:**

Of 5,389 cases reported to MOH during the study period, 1,754 (32.5%) were asymptomatic. Amongst symptomatic persons 2,747 (75.6%) had mild COVID-19 and 888 (24.4%) had moderate-to-severe disease; the most common symptoms were fever (69.6%), cough (51.9%), and myalgia (45.8%). The odds of having moderate-to-severe disease were highest among persons with hypertension (aOR = 1.72, 95% Confidence Interval [CI] (1.34, 2.20), chronic pulmonary disease (aOR = 3.93, 95% CI (1.93, 8.17)) and tuberculosis (aOR = 3.44, 95% CI (1.35, 9.14)) compared to persons without those conditions. The odds of having moderate-to-severe disease increased with age but was also seen among children aged 0–4 years (OR: 1.73, 95% CI (0.93, 3.08)), when using 30–39 years old as the reference group. All of the older age groups, 50–64 years, 65–74 years, 75–84 years, and 85+ years, had significantly higher odds of having moderate-to-severe COVID-19 compared with ages 30–39 years. Diabetes was associated with elevated odds of moderate-to-severe disease in bivariate analysis (OR = 2.17, 95% CI (1.58,2.98) but, this association did not hold in multivariable analyses (aOR = 1.22,95%CI (0.86,1.72)).

**Conclusion:**

These findings from a resource-constrained country highlight the importance of surveillance systems to track emerging infections and their risk factors. In addition to co-morbidities described elsewhere, tuberculosis was a risk factor for moderate-to-severe COVID-19 disease.

## Introduction

In December 2019, a new infectious disease with pulmonary symptoms, caused by the severe acute respiratory syndrome coronavirus 2 (SARS-CoV-2) was observed in China [1]. On January 30, 2020, the World Health Organization (WHO) declared this new coronavirus disease to be a “public health emergency of international concern” and stated that early detection, isolation and case treatment, contact tracing, and social distancing measures should be applied to curb its dissemination [2]. By March 6, 2021, 115,641,975 cases were reported in 222 countries with 2,571,067 deaths [3], and the pandemic is ongoing as of June 2021.

**Fig 1:**
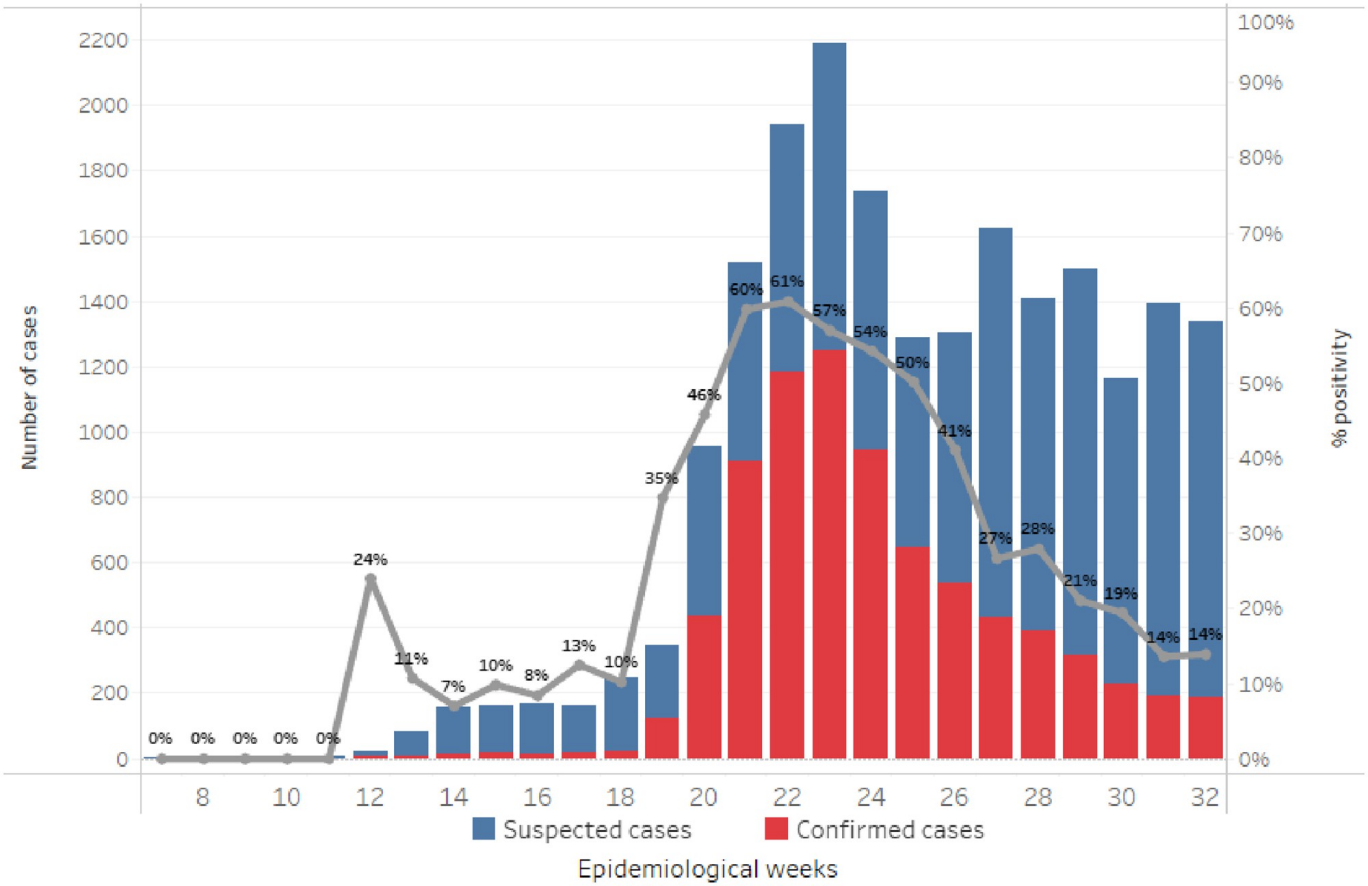
SARS-CoV-2 outbreak by week of onset–Haiti: Number of suspected and confirmed outpatient SARS-CoV-2 cases and percent positivity of tests by epidemiological week from March 18 to August 4, 2020.

In January 2020, as soon as reports of cases of COVID-19 outside of China were confirmed, the Haitian Ministry of Public Health and Population (MSPP) started adapting surveillance and emergency preparedness measures. Among public health measures to prevent the introduction of the virus, MSPP implemented a screening system at the points of entry where all international travelers were systematically screened for COVID-19 symptoms, suspected cases tested and isolated, and contacts quarantined. As a result, on March 18, 2020, Haiti confirmed its first two COVID-19 cases, both among persons arriving from Europe. From the first confirmed cases in March 2020, Haiti saw a steep increase in the number of SARS-CoV-2 test positive persons in all ten departments (equivalent of a region; first-level administrative subdivision) beginning in early May 2020 and reaching a peak during intense community transmission later that month [4] [Fig 1]. At the peak of the outbreak, SARS-CoV-2 RT-PCR and molecular test positivity rates as high as 61% were observed and declined with the expanded availability of testing to reach around 9% by August 8, 2020 [Fig 1]. These high SARS-CoV-2 test positivity rates could be explained by testing priority given to symptomatic people and immediate contacts of those who tested positive, due to limited testing capacity. Cases rose to 7,544 with 171 deaths and 2.26% case fatality rate by August 4, 2020 [4].

Clinical manifestations and outcomes of COVID-19 differ across countries [5]. The main objective of this study was to describe clinical manifestations of COVID-19 among Haitian outpatients and to identify risk factors for moderate to severe COVID-19 disease, in order to better inform public health interventions. In this report we describe the characteristics of outpatients diagnosed with COVID-19 and the risk factors associated with the severity of their clinical manifestations.

## Methods

### Ethical considerations

The protocol was approved by the National Bioethical Committee of Haiti on June 8, 2020 (Ref-1920–34). The activity was reviewed by CDC and was conducted consistent with applicable federal law and CDC policy (45 C.F.R. part 46, 21 C.F.R. part 56; 42 U.S.C. §241(d); 5 U.S.C. §552a; 44 U.S.C. §3501 et seq). The informed consent requirement was waived due to the retrospective design of this investigation.

### Surveillance system

**Fig 2:**
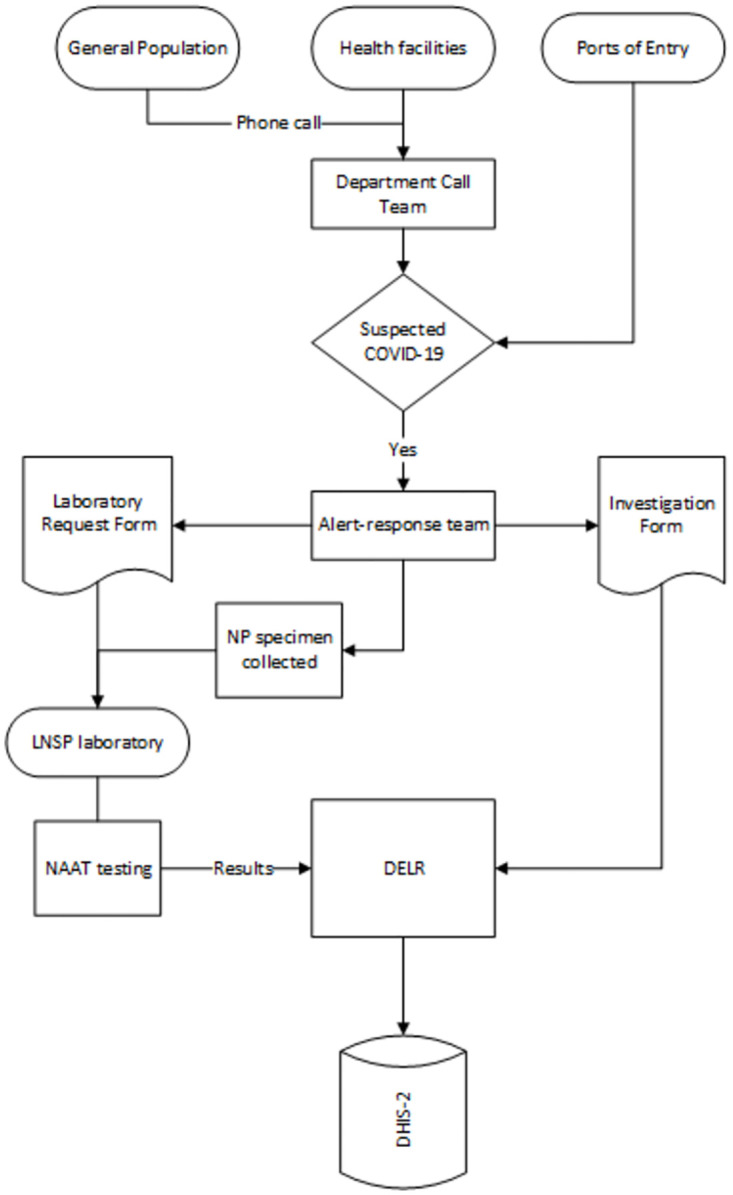
Surveillance flow chart for reporting outpatient SARS-CoV-2 cases—Haiti: Data streams for the detection, investigation, and testing of persons suspected of having SARS-CoV-2 infection from March 18 to August 4, 2020. NP: Nasopharyngeal; LNSP: National Public Health Laboratory (*Laboratorie nationale de santé publique*; NAAT: Nucleic acid amplification testing; DELR: Directorate of Epidemiology, Laboratories and Research (*Direction d’epidemiologie*, *de laboratoire et de recherches*).

In January 2020, the Ministry of Health (MOH) through its Directorate of Epidemiology, Laboratories and Research (*Direction d’epidemiologie*, *de laboratoire et de recherches*—DELR) established a dynamic system to identify and investigate suspected cases of SARS-CoV-2 infection. Haiti used DELR’s National Epidemiologic Surveillance Network (*Réseau nationale de surveillance épidemiologique*—RNSE) to gather surveillance data from three main sources: alerts from the general population, health facilities in all ten departments (equivalent of region), and active screening at border/ports of entry (Fig 2).

Members of the general public or healthcare providers (e.g. The Red Cross and private practitioners) were directed to “alert” DELR of any persons who had signs or symptoms consistent with COVID-19 disease (e.g. fever, cough, asthenia, dyspnea and diarrhea), by calling a department-specific hotline. In each department, a “Call Team” received alerts about suspected COVID-19 cases, conducted a preliminary screening of cases, and relayed details of suspected cases to their respective Alert-Response teams at DELR or departmental level representatives of the MOH. The Alert-Response team, \comprising epidemiologists, epidemiologic surveillance officers, nurses or laboratory technicians, confirmed that suspect cases met the case definition for COVID-19 and initiated a case investigation at the individual’s home within 24 hours of the notification. All symptomatic suspected cases and laboratory confirmed COVID-19 cases were isolated at home or at one of the government quarantine institutions. The Alert-Response team used tablets loaded with the RNSE application to complete the investigation form, including demographic information, clinical signs/symptoms, and co-morbidities. Co-morbidity status was assessed by self-report. Then, the Alert-Response team conducted contact tracing for all confirmed cases.

Among close contacts of confirmed cases, the Alert-Response team completed an investigation form and instructed the contact(s) to quarantine at home. They followed each close contact daily to monitor their body temperature and for the development of COVID-19 symptoms during the 14 days of quarantine. Suspect cases among contacts who exhibited a body temperature higher than 38°C (fever) or any other symptoms related to COVID-19 were referred for SARS-CoV-2 testing. All close contacts were tested for SARS-CoV-2 on day twelve of quarantine or at symptom onset. If the test result was negative, and no further symptoms were present within the 14-day period, restrictions on the individual were lifted.

In parallel, MSPP established a surveillance system at the borders and ports of entry to identify and isolate suspected COVID-19 cases arriving from abroad. The DELR Alert-Response team completed an investigation form for each traveler who arrived at the ports of entry or borders, including screening questions for COVID-19 symptoms and body temperature monitoring. All persons entering Haiti were considered suspect cases and were quarantined at home or at one of the government quarantine institutions for fourteen days per established protocols. The Alert-Response team followed the same guidelines as indicated above for all close contacts of confirmed cases from the general population.

Oral and nasopharyngeal swabs were collected by the Alert-Response team for all suspect cases and their close contacts. Case investigation and laboratory data were transmitted to the District Health Information Software (DHIS2, version 2.0) electronic platform through a departmental data management team (Fig 2).

The diagnosis of COVID-19 followed WHO interim guidance [6]. Confirmed cases of COVID-19 were determined through a nucleic acid amplification test (NAAT) using reverse transcriptase polymerase chain reaction (RT-PCR).

### Inclusion and exclusion criteria

Any non-hospitalized person with a positive reverse transcriptase polymerase chain reaction (RT-PCR) on a nasopharyngeal and oropharyngeal sample from March 18, 2020 to August 4, 2020 was included in the data regardless of nationality or country of origin. Hospitalized persons with COVID-19 were excluded from this analysis because inpatient data from COVID-19 treatment centers weren’t automatically transmitted to the DHIS2 platform during the study period. Any person with symptoms consistent with COVID-19 who had a RT-PCR negative or lacked a confirmatory RT-PCR test was excluded.

### COVID-19 RT-PCR laboratory sample collection, storage, transportation, and testing methods

After collection of oral or nasopharyngeal swabs at the health facilities or at the patient’s home, the sample was stored at a temperature between 2 and 8 degrees centigrade and transported to the National Public Health Laboratory (*Laboratorie nationale de santé publique*–LNSP) for testing. Samples were tested using a RT-PCR protocol as described previously [7].

### Case definitions

According to definitions established by DELR [4], a suspected COVID-19 case was defined as someone fulfilling any one of the following criteria: 1) anyone with a temperature higher than 38 degrees Celsius, or a recent history of fever (in the last 14 days), with or without the presence of cough, difficulty breathing, body aches, or unexplained headache; 2) anyone coming from an area/foreign country at risk of COVID-19 and/or having an epidemiologic link with a confirmed COVID-19 case; or 3) any symptomatic person who does not meet other epidemiological criteria with a positive rapid diagnostic test for antigens (RDT-Ag) of SARS-CoV-2.

A confirmed case of COVID-19 was defined as anyone with a positive SARS-CoV-2 NAAT, irrespective of symptoms.

A close contact is defined as: 1) anyone residing in the same household as a COVID-19 case; 2) anyone who has had direct physical contact with a COVID-19 case for example, shaking hands; 3) or anyone with direct unprotected contact with infectious secretions from a COVID-19 case (e.g. health workers).

### Data extraction and management

Data for laboratory-confirmed COVID-19 cases that were reported from March 18, 2020, to August 4, 2020, were extracted from DHIS2. Data extracted included demographic and epidemiological data, the presence of COVID-19 signs and symptoms, and underlying co-morbidities.

In our study, symptomatic cases were classified into two disease severity groups ˗ mild and moderate-to-severe—based on their clinical presentation. A confirmed case that presents with any of the following signs or symptoms, such as: fever, cough, myalgia, headache, asthenia, ageusia, anosmia, diarrhea was classified as mild case; a moderate-to-severe outpatient case was defined as a case with the presence of dyspnea. Asymptomatic or pre-symptomatic cases were those with positive tests but without symptoms and not classified into the two groups.

### Statistical analysis

Statistical analyses were conducted using R software (RStudio v3.6.2, Free Software Foundation Inc., Boston, MA) and Statistical Analysis System (SAS) software (SAS Institute, Cary, NC; version 9.4). Frequency procedures were used to generate descriptive statistics. Chi-Square (χ^2^) test statistics or Fisher’s exact test were used to test association of sex and age categories of non-hospitalized COVID-19 cases by the severity of the disease, as well as by asymptomatic/symptomatic. Mood’s median test was used to compare age medians between the mild and moderate-to-severe cases as well as the asymptomatic and symptomatic cases.

Further, we ran bivariate logistic regression to understand the association of predictors and having moderate-to severe COVID-19 over mild COVID-19. Bivariate logistic regression was conducted with COVID-19 severity as the binary outcome against each of the following variables: age, sex, quarantine status, co-morbidities (i.e., cancer, hypertension, diabetes, kidney disease, chronic pulmonary disease, and tuberculosis), and signs or symptoms type (i.e., fever, cough, myalgia, headache, asthenia, ageusia, anosmia, diarrhea).

Then, we examined the association between severity of COVID-19 and comorbidities, while adjusting for other variables using multivariable logistic using least absolute shrinkage and selection operator regression (LASSO) for covariate selection. LASSO is a shrinkage method, meaning that it shrinks “weak” beta values, or coefficients, towards zero. If nonzero, they are selected. LASSO chooses a subset of predictors by introducing an upper bound for the sum of squares, hence minimizing the errors present in the model. LASSO reduces collinearity and increases precision. The goal of using this is to find the covariates, or risk factors/comorbidities in our case, to see what is selected, what the coefficients are, and interpret them [8]. Selected variables were put in the final multivariable logistic regression models to generate adjusted odds ratios (aORs).

## Results

### Demographic and clinical data

From March 18 to August 4, 2020, a total of 5,389 persons who tested positive for SARS-CoV-2 by RT-PCR were reported to MOH. The median age of symptomatic persons was 38 years (IQR: 30–50 years) and 60.0% (n = 3,235) were male (Table 1). Among COVID-19 cases, 1,754 (32.5%) were classified as asymptomatic and 3,635 (67.5%) were symptomatic. The median age between asymptomatic and symptomatic cases was different with the median for asymptomatic being 37 years old and the median for symptomatic being 38 years old (p-value = 0.024 from Mood’s median test). There was a significant difference for sex by asymptomatic and symptomatic cases (p-value = 0.018 from Chi-square test). Among COVID-19 cases, co-morbidities that were more common than others in patients were hypertension (7.2%), diabetes (3.3%), and HIV (1.5%) (Table 1).

**Table 1:** Characteristics of all COVID-19 cases, by symptom status, Haiti, March to August 2020.

Characteristics	All cases (n = 5,389)	Symptomatic (n = 3,635)	Asymptomatic (n = 1,754)
**Age, median (IQR)**	38 (30–50)	38 (30–51)	37 (29–48)
p-value = 0.024*			
**Age groups (years)**			
p-value<0.001†			
0–4	72 (1.3)	57 (79.2)	15 (20.8)
5–9	32 (0.6)	19 (59.4)	13 (40.6)
10–14	48 (0.9)	23 (47.9)	25 (52.1)
15–29	1163 (21.6)	763 (65.6)	400 (34.4)
30–39	1649 (30.6)	1096 (66.5)	553 (33.5)
40–49	1018 (18.9)	671 (65.9)	347 (34.1)
50–64	894 (16.6)	629 (70.4)	265 (29.6)
65–74	319 (5.9)	232 (72.7)	87 (27.3)
75–84	142 (2.6)	103 (72.5)	39 (27.5)
85+	52 (1.0)	42 (80.8)	10 (19.2)
**Sex**			
p-value = 0.018†			
Male	3235 (60.0)	2142 (66.2)	1093 (33.8)
Female	2154 (40.0)	1493 (69.3)	661 (30.7)
**Comorbidities** **			
Cancer	11 (0.2)	11 (100)	0 (0.0)
Hypertension	398 (7.2)	370 (93.0)	28 (7.0)
Diabetes^††^	180 (3.3)	169 (93.9)	11 (6.1)
HIV	79 (1.5)	62 (78.5)	17 (21.5)
Kidney Disease	23 (0.4)	20 (87.0)	3 (13.0)
Malnutrition	7 (0.1)	7 (100)	0 (0.0)
Liver Cirrhosis	8 (0.1)	8 (100)	0 (0.0)
Chronic Pulmonary Disease	43 (0.8)	39 (90.7)	4 (9.3)
Tuberculosis	24 (0.4)	23 (95.8)	1 (4.2)
None	4,756 (88.3)	3057 (64.3)	1699 (35.7)
**Others**			
Patient quarantined	1743 (33.0)	1218 (69.9)	525 (30.1)

HIV, human immunodeficiency virus.

Data are n (%), and median (IQR).

* The non-parametric p-value is calculated by Mood’s median test. P <0.05 was considered statistically significant.

^†^ The parametric p-value is calculated by Mantel-Haenszel chi-square test with Bonferroni adjustment for age categories. P <0.05 was considered statistically significant. The p-value represents the difference between the asymptomatic and symptomatic.

^††^Diabetes co-morbidity did not distinguish between Type I and Type II

^§^ Vertigo, paresthesia, tremor

** This does not equal 100% because some people could have multiple comorbidities

Table 2 describes characteristics of symptomatic cases, specifically comparing mild and moderate-to-severe cases. Among symptomatic persons, fever was the most common sign/symptom (n = 2,529, 69.6%), followed by cough (n = 1,885, 51.9%), and myalgia (n = 1,664, 45.8%) (Table 2). The median ages for mild and moderate-to-severe were 37 and 44 years old, respectively. Patients with mild disease were slightly younger than those with moderate-to-severe disease (p<0.05 from Mood’s median test); but sex did not significantly differ by severity of symptoms (p-value = 0.519 from Chi-square test). Almost all comorbidities showed higher numbers of patients in the mild disease except for chronic pulmonary disease and TB that had higher number of patients in the moderate-to-severe disease.

**Table 2:** Characteristics of symptomatic COVID-19 cases by disease severity.

Characteristics	Symptomatic (n = 3,635)	Moderate-to-Severe (n = 888)	Mild (n = 2,747)
**Age, median (IQR)**	38 (30–51)	44 (32–60)	37 (30–48)
p-value<0.001*			
**Age groups (years)**			
p-value<0.001†			
0–4	57 (1.6)	16 (28.1)	41 (71.9)
5–9	19 (0.5)	5 (26.3)	14 (73.7)
10–14	23 (0.6)	2 (8.7)	21 (91.3)
15–29	763 (21.0)	159 (20.8)	604 (79.2)
30–39	1096 (30.2)	202 (18.4)	894 (81.6)
40–49	671 (18.5)	148 (22.1)	523 (77.9)
50–64	629 (17.3)	181 (28.8)	448 (71.2)
65–74	232 (6.4)	104 (44.8)	128 (55.2)
75–84	103 (2.8)	48 (46.6)	55 (53.4)
85+	42 (1.2)	23 (54.8)	19 (45.2)
**Sex**			
p-value = 0.519†			
Male	2142 (58.9)	356 (23.8)	1,137 (76.2)
Female	1493 (41.1)	532 (24.8)	1,610 (75.2)
**Comorbidities** **			
Cancer	11 (0.3)	4 (36.4)	7 63.6)
Hypertension	370 (10.2)	159 (43.0)	211 (57.0)
Diabetes^††^	169 (4.6)	68 (40.2)	101 (59.8)
HIV	62 (1.7)	16 (25.8)	46 (74.2)
Kidney Disease	20 (0.6)	4 (20.0)	16 (80.0)
Malnutrition	7 (0.2)	3 (42.9)	4 (57.1)
Liver Cirrhosis	8 (0.2)	3 (37.5)	5 (62.5)
Chronic Pulmonary Disease	39 (1.1)	22 (56.4)	17 (43.6)
Tuberculosis	23 (0.6)	13 (56.5)	10 (43.5)
None	3057 (84.1)	659 (21.6)	2389 (78.4)
**Clinical signs/symptoms**			
Fever	2529 (69.6)	663 (26.2)	1,866 (73.8)
Cough	1885 (51.9)	568 (30.1)	1,317 (69.9)
Myalgia	1664 (45.8)	442 (26.6)	1,222 (73.4)
Headache	1463 (40.2)	350 (23.9)	1,113 (76.1)
Asthenia	1271 (35.0)	402 (31.6)	869 (68.4)
Ageusia	1157 (31.8)	287 (24.8)	870 (75.2)
Anosmia	1006 (27.7)	237 (23.6)	769 (76.4)
Dyspnea^¶^	888 (24.4)	888 (100.0)	0 (0.0)
Diarrhea	347 (9.5)	126 (36.3)	221 (63.7)
Other neurologic symptoms^§^	7 (0.2)	2 (28.6)	5 (71.4)
**Others**			
Patient quarantined	1218 (33.5)	378 (31.0)	840 (69.0)

HIV, human immunodeficiency virus.

Data are n (%), and median (IQR).

*The non-parametric p-value is calculated by Mood’s median test. P <0.05 was considered statistically significant.

^†^ The parametric p-value is calculated by Mantel-Haenszel chi-square test with Bonferroni adjustment for age categories. P <0.05 was considered statistically significant. The p-value represents the difference between the asymptomatic and symptomatic.

^††^Diabetes co-morbidity did not distinguish between Type I and Type II

^§^ Vertigo, paresthesia, tremor

** This does not equal 100% because some people could have multiple comorbidities

We also conducted a risk factor analysis with bivariate and multivariable logistic regression models with disease severity as the outcome. Variables that were selected by LASSO regression for the multivariable models are listed with odds ratios under their respective model (Table 3). These analyses only include symptomatic cases and correspond to the numbers from Table 2.

**Table 3:** Logistic regression modelling evaluating risk factors related to the severity of clinical manifestations of COVID-19 outpatients, i.e. comparing moderate-to-severe and mild case as the outcome.

Characteristics	Bivariate logistic regression			Multivariable lasso regression		
	OR	(95%CI)	p-value	aOR	(95%CI)	p-value
**Age groups (years)**						
0–4	1.73	(0.93, 3.08)	0.073	1.64	(0.88, 2.97)	0.102
5–9	1.58	(0.51, 4.18)	0.385	1.59	(0.51, 4.22)	0.378
10–14	0.43	(0.06, 1.45)	0.246	0.44	(0.07, 1.51)	0.268
15–29	1.17	(0.92, 1.47))	0.197	1.16	(0.92, 1.55)	0.203
30–39	(ref)	-	-	ref	-	-
40–49	1.25	(0.99, 1.59)	0.0637	1.22	(0.96, 1.55)	0.104
50–64	1.79	(1.42, 2.25)	<0.001	1.58	(1.24, 2.00)	<0.001
65–74	3.60	(2.66, 4.86)	<0.001	2.97	(2.16, 4.06)	<0.001
75–84	3.86	(2.54, 5.86)	<0.001	2.98	(1.92, 4.58)	<0.001
85+	5.36	(2.86, 10.12)	<0.001	3.72	(1.94, 7.20)	<0.001
**Sex**						
Male	1.05	(0.90, 1.23)	0.493	-	-	-
Female	(ref)			(ref)		
**Comorbidities**						
Cancer	1.77	(0.38, 6.98)	0.363	-	-	-
Hypertension	2.62	(2.10, 3.27)	<0.001	1.72	(1.34, 2.20)	<0.001
Diabetes^††^	2.17	(1.58, 2.98)	<0.001	1.22	(0.86, 1.72)	0.264
HIV	1.08	(0.59, 1.87)	0.799	-	-	-
Kidney Disease	0.77	(0.19, 2.40)	0.645	0.58	(0.14, 1.81)	0.397
Malnutrition	2.32	(0.34, 13.77)	0.270	-	-	-
Liver Cirrhosis	1.86	(0.38, 7.59)	0.397	0.24	(0.03, 1.51)	0.144
Chronic Pulmonary Disease	4.08	(2.16, 7.82)	<0.001	3.93	(1.93, 8.17)	<0.001
Tuberculosis	4.07	(1.78, 9.56)	<0.001	3.44	(1.35, 9.14)	0.010
**Clinical signs/symptoms**						
Fever	1.39	(1.17, 1.65)	0.002	-	-	-
Cough	1.93	(1.65, 2.25)	<0.001	-	-	-
Myalgia	1.24	(1.06, 1.44)	0.006	-	-	-
Headache	0.95	(0.82, 1.11)	0.560	-	-	-
Asthenia	1.79	(1.53, 2.09)	<0.001	-	-	-
Ageusia	1.03	(0.87, 1.21)	0.718	-	-	-
Anosmia	0.94	(0.79, 1.11)	0.450	-	-	-
Diarrhea	1.89	(1.49, 2.38)	<0.001	-	-	-
Other neurologic symptoms^§^	1.24	(0.24, 6.39)	0.681	-	-	-

ref, reference group; HIV, human immunodeficiency virus

^††^Diabetes co-morbidity did not distinguish between Type I and Type II

^§^ Vertigo, paresthesia, tremor

In bivariate regression, using persons aged 30–39 years as the reference group, we see higher odds of having moderate-to-severe disease among adults aged 50 years and older, with adults aged ≥85 years at the highest odds of moderate-to-severe disease (OR: 5.36; 95% CI:2.86, 10.12). Of the comorbidities, persons with hypertension (OR: 2.62; 95% CI: 2.10, 3.27), diabetes (OR: 2.17; 95% CI: 1.58, 2.98), chronic pulmonary disease (OR: 4.08; 95% CI: 2.16, 7.82), and tuberculosis (OR: 4.07; 95% CI: 1.783, 9.56) had higher odds of having moderate-to-severe COVID-19 disease than those without those conditions. Of the symptoms, persons with fever (OR: 1.39; 95% CI: 1.17, 1.65), cough (OR: 1.93; 95% CI: 1.65, 2.25), myalgia (OR: 1.24; 95% CI: 1.06, 1.44), asthenia (OR: 1.79; 95% CI: 1.53, 2.09), and diarrhea (OR: 1.89; 95% CI: 1.49, 2.38) had higher odds of having moderate-to-severe COVID-19 disease than those without those conditions.

In the multivariable model including age, hypertension, diabetes, kidney disease, liver cirrhosis, chronic pulmonary disease, and tuberculosis, odds of moderate-to-severe COVID-19 was greater than the reference group among persons aged 50–64 years and progressively increased among successively older age groups (Table 3).

## Discussion

To our knowledge, this is the first report about the clinical characteristics and risk factors for moderate-to-severe COVID-19 among outpatients in Haiti. Knowledge about clinical manifestations in the context of an emerging disease is important not only to provide clinicians with accurate tools to better diagnose potential for moderate- to-severe disease, but also to establish evidence-based case definitions for surveillance and improve management of COVID-19 disease. Most published clinical studies examining COVID-19 emerge from Asia, Europe, and the United States [9–11]. Emergent research appears to overlook underlying clinical characteristics of COVID-19 involving low- and middle-income country populations [12].

As has been seen in other settings, age was an important risk factor for COVID-19 disease severity among Haitians [9, 13]. Progressively increasing odds of moderate-to-severe disease were seen in the age groups greater than 50 years of age. This aligns with results of a large study involving half a million COVID-19 patients which revealed that age is positively associated with case fatality rates with a cutoff age of 50 years [9].

Our study results showed that fever and cough are the predominant symptoms among COVID-19 cases, highlighting the need for clinicians to consider SARS-CoV-2 as an etiologic agent when these symptoms are present. Some studies reported in children cough or loss of a sense of taste less frequently than in adults [14, 15]. The contributions of these signs and symptoms in countries with limited testing capacity should be assessed in further studies as COVID-19 testing is a major challenge for developing countries like Haiti.

Cardiovascular disease was an important factor related to COVID-19 severity among our study population. Our study showed an increased odds of moderate-to-severe COVID-19 in cases with hypertension; however evidence on the role of hypertension as a risk factor for COVID-19 across settings is mixed [16, 17]. While diabetes was associated with elevated odds of moderate-to-severe disease in bivariate analysis, this association did not hold in multivariable analyses. This absence of association with COVID-19 is intriguing as diabetes is often considered a risk factor for disease severity and mortality, which calls for further investigation of the relationship between diabetes and COVID-19 among Haitians [18, 19]. Literature has shown that while diabetes is associated with severe and fatal COVID-19, the highest risk of severe COVID-19 outcomes are seen among persons presenting with metabolic syndrome, assessed by using the triad of hypertension diabetes, and obesity as a proxy for this condition [20–22].

Chronic pulmonary disease also played an important role in disease severity in our study. The increased odds of moderate-to-severe COVID-19 in cases with tuberculosis and chronic pulmonary disease is not surprising, as COVID-19 often manifests as pneumonia and other respiratory symptoms. More limited and mixed data exists on the impact of tuberculosis infection on COVID-19 outcomes. However, many of these studies originate from middle and high-income countries where tuberculosis is less frequent and disease outcomes may be different [23, 24]. It is possible that infection with SARS-CoV-2 causes a temporary suppression of cellular immunity which further predisposed our population to aggravated reactivation or new infection with tuberculosis [25].

The data show that a third of our study population who were positive for SARS-CoV-2 were asymptomatic. This highlights the efforts of the MOH to identify and isolate COVID-19 cases that may curb the propagation of COVID-19. Mathematical models have shown that contact tracing is useful to decrease the expansion of coronavirus in a population. The sooner a contact tracing system is established, the greater the impact of infection prevention [26–30]. Evidence suggests that a portion of persons who are asymptomatic at the time of testing have viral loads that may lead to onward transmission [31–32]. While persons without symptoms may have lower transmission potential [33–34], nonetheless, they may represent an important source of SARS-CoV-2 transmission with approximately 50% of transmission linked to persons without symptoms (either pre-symptomatic or asymptomatic individuals) [35]. Hence, it is important to increase testing availability in Haiti, to be able to quickly identify and isolate COVID-19 cases.

This investigation was subject to several limitations. We did not follow our study population and, hence, we cannot affirm whether they were truly asymptomatic or pre-symptomatic with latent symptoms. Furthermore, as our study population did not have chest imaging, it is possible that some that we classified as asymptomatic or mild could have been classified as moderate-to-severe if chest imaging were available and some abnormalities were present on their imaging according to the NIH classification [36]. An important limitation of this study stems from self-declaration of comorbidities. This may induce a bias towards the null of the prevalence of hypertension and diabetes, particularly since these comorbidities may not show overt clinical symptoms and many individuals may be undiagnosed as access to health care remains limited in Haiti. Furthermore, some potentially relevant risk factors, namely overweight or obesity status, were not collected due to limited feasibility of measuring height and weight in a field setting. Another limitation is that this study does not provide information about laboratory parameters, radiologic abnormalities, and case follow-ups. To gain a better understanding of COVID-19 patient profiles, more detailed information on cases, particularly paraclinical examinations and clinical outcomes, should be collected in future studies. Another important limitation is the fact that SARS-CoV-2 testing priority was given to symptomatic individuals and immediate contacts of those who tested positive, due to limited testing capacity of the MOH. This explained the high proportion of symptomatic infection among COVID-19 positive individuals, and this highlights an urgent need for increasing access to SARS-CoV-2 testing in Haiti. Finally, this study only included non-hospitalized patients, which potentially skewed some odds ratios towards the null, as people with moderate-to-severe symptoms may be more likely to be hospitalized.

This report highlighted critical information that could help the Haitian Ministry of Health and the health care professionals to assist with the identification and management of COVID-19 in Haiti. Further, these data may contribute to determining which groups may be prioritized in the initial phase of the COVID-19 vaccination program in Haiti, when available. Also, this highlights the importance of screening for and management of chronic pulmonary disease, hypertension, and tuberculosis as we have seen that these comorbidities may increase the risk of severity of clinical manifestations of SARS-CoV-2 infection and thus could help achieve better health outcomes from COVID-19. The findings in this report highlight the importance of management and control of chronic conditions in countries such as Haiti.
